# Reagent-Free Colorimetric Assay for Galactose Using Agarose Gel Entrapping Nanoceria and Galactose Oxidase

**DOI:** 10.3390/nano10050895

**Published:** 2020-05-08

**Authors:** Phuong Thy Nguyen, Hee Tae Ahn, Moon Il Kim

**Affiliations:** Department of BioNano Technology, Gachon University, 1342 Seongnamdae-ro, Sujeong-gu, Seongnam, Gyeonggi 13120, Korea; nnphuongthy18@gmail.com (P.T.N.); venice4@naver.com (H.T.A.)

**Keywords:** reagent-free colorimetric assay, galactose determination, nanoceria, agarose gel, galactosemia diagnosis

## Abstract

A reagent-free colorimetric method for galactose quantification using a composite of cerium oxide nanoparticles (nanoceria) and galactose oxidase (Gal Ox) entrapped in an agarose gel was developed. In the presence of galactose, the Gal Ox entrapped within the agarose gel catalyzed the oxidation of galactose to generate H_2_O_2_, which induced a color change from white to intense yellow. This reaction occurred without any chromogenic substrate. This color transition is presumed to be due to the H_2_O_2_-mediated alteration of the oxidation state of cerium ions present on the surface of the nanoceria. The intensity of color change was quantified by acquiring an image with a conventional smartphone, converting the image to cyan-magenta-yellow-black (CMYK) mode, and subsequently analyzing the image using the ImageJ software. Using this strategy, galactose concentration was specifically determined with excellent sensitivity of as low as 0.05 mM. The analytical utility of the assay was successfully verified by correctly determining diverse levels of galactose in human serum, which is enough to diagnose galactosemia, a genetic disorder characterized by the malfunctioning of enzymes responsible for galactose metabolism. The assay employing a hydrogel composite with entrapped nanoceria and Gal Ox, is a simple, cost-effective, and rapid colorimetric assay for galactose quantification, without using any chromogenic reagent. This cost-effective method has great potential for the diagnosis of galactosemia and is highly promising in comparison to the laborious instrumentation-based methods currently in use.

## 1. Introduction

Galactose, one of the breakdown products of lactose (a disaccharide composed of one glucose and one galactose molecule) in milk, is an essential nutrient in the human body. It is also an important marker for diagnosing a disease that affects newborns, galactosemia. Galactosemia is a congenital metabolic disorder caused by the deficiencies of relevant enzymes involved in galactose metabolism [1]. Galactosemia is categorized into types I, II, and III, which occur due to the malfunctioning of galactose-1-phosphate uridyltransferase (GALT), galactokinase (GALK), and uridine diphosphate galactose-4-epimerase (GALE), respectively [2,3]. Among these, type I galactosemia is recognized as the most common and severe form of galactosemia and is termed “classic galactosemia” [4]. Patients with classic galactosemia cannot metabolize galactose and galactose-1-phosphate, leading to their accumulation in blood, and thus causing life-threatening complications, such as jaundice, cataracts, hepatic failure, and kidney dysfunction [5,6]. Thus, screening newborns for galactosemia, by evaluating the levels of galactose and galactose-1-phosphate or the efficiency of relevant enzymes in the blood, is crucial for its early diagnosis and effective treatment.

Many analytical methods have been developed for the diagnosis of galactosemia using dried blood spot specimens of newborns. The most traditional method is based on bacterial growth inhibition, as suggested by Paigen et al. [7]. This assay uses a mutant *Escherichia coli* strain to check its resistance or sensitivity to bacteriophage infection in the presence or absence of galactose. The bacteria are resistant to bacteriophage infection in the presence of galactose, whereas they are sensitive and are infected by bacteriophages in its absence. This method quantifies galactose levels by measuring the bacterial growth zone around the blood spots. Other methods employing genetically-engineered bacteria have also been reported [8]. For example, a transferase-deficient *E. coli* mutant whose growth is inhibited by galactose was employed for galactose quantification and the subsequent diagnosis of galactosemia. A galactose-1-phosphate uridylyltransferase-encoding gene (*galT*) knockout strain of *E. coli* exhibited growth proportional to glucose levels but not galactose levels, while another *E. coli* strain grew normally in the presence of either glucose or galactose. Galactose concentrations were quantified by measuring the growth differences between the two strains. All these microbiological methods can detect galactose with high specificity; however, they generally require long incubation times for bacterial growth. Additionally, microbial strains are sometimes unstable, yielding inaccurate results, leading to repetitive testing to measure galactose levels more accurately and exclude any false positive results. Currently, galactose levels are usually determined using diverse analytical instruments, such as high-performance liquid chromatography (HPLC), gas chromatography/mass spectrometry (GC/MS), and tandem mass spectrometry (MS/MS) [9,10,11]. Assays employing these techniques enable sensitive and accurate analyses for galactose, but are expensive and often complicated to operate due to many pre-/post-treatment steps; hence, they are not suitable for rapid screening of newborns.

A microscale well-plate assay based on a coupled enzymatic reaction involving alkaline phosphatase and galactose dehydrogenase was more recently developed [12]. In this assay, alkaline phosphatase catalyzes the production of galactose from galactose-1-phosphate in the presence of galactose dehydrogenase and NAD^+^ to yield NADH. The resulting NADH activates diaphorase to convert iodonitrotetrazolium violet into a red colored product, formazan, which can be visually detected. Another reported cascade enzyme reaction utilizes a multi-catalyst system composed of Gal Ox and a peroxidase mimicking nanozyme [13]. In the presence of galactose, Gal Ox generates H_2_O_2_, which subsequently activates peroxidase-mimicking magnetic nanoparticles and converts a colorimetric substrate of 2,2′-azino-bis(3-ethylbenzo-thiazoline-6-sulfonic acid) diammonium salt (ABTS) into a green product. These enzymatic methods are quite sensitive and quantitative, but multiple components, including the colorimetric substrate and complicated procedures, still limit their widespread use. These enzymes have been also widely employed to develop electrochemical assays, which could yield the highest sensitivity with the lowest detection limits without the requirement of pre-/post-treatment steps [14]. Some of them based on the unique screen-printed electrode and Langmuir–Blodgett film-deposited electrode showed impressive detection limits, even down to the low nanomolar level; however, the appropriate electrodes and electrometer should be required for the measurement of galactose level [15,16]. Thus, there is a great incentive to develop a simpler, more rapid, and more economical method for galactose determination.

Recently, we reported the use of a multi-catalyst system consisting of glucose oxidase or cholesterol oxidase in combination with nanoceria as a colorimetric strategy that helps with detecting the corresponding target molecules [17]. This assay involves an H_2_O_2_-mediated color change of nanoceria without any addition of colorimetric reagents; however, it still depends on spectrophotometry for quantification. This feature critically hinders its widespread applications, particularly in facility-limited environments. As an advancement of this work for practical on-site application, we herein developed a composite system, which consists of nanoceria and Gal Ox as an oxidative enzyme within an agarose gel. Essential analytical features of the system, such as selectivity, sensitivity, and detection precision, can be assessed using images acquired with a smart cellular phone, which is quite suitable for instrumentation-free, point-of-care testing (POCT) environments.

## 2. Experimental Section 

### 2.1. Materials

Nanoceria (<5 nm particle size), agarose (low-gelling temperature), galactose oxidase from *Dactylium dendroides* (Gal Ox), galactose, arabinose, fructose, lactose, maltose, glucose, sodium acetate, and human serum were purchased from Sigma-Aldrich (Milwaukee, WI, USA). The average particle size of the nanoceria was checked by transmission electron microscopy (TEM) analysis. Hydrogen peroxide (35%) was purchased from Junsei Chemical Co. (Tokyo, Japan). All the chemicals were of analytical grade or higher, and all solutions were prepared with distilled (DI) water purified using a Milli-Q Purification System (Millipore, Billerica, MA, USA).

### 2.2. Preparation of Agarose Gel Composites Entrapping Nanoceria Only (Agarose_Nanoceria) and Both Gal Ox and Nanoceria (Agarose_Nanoceria + Gal Ox)

Agarose gel composites were synthesized by slightly modifying the reported procedure [17]: 2% (*w*/*v*) agarose solution was first prepared by dissolving agarose in distilled water using microwave treatment for 1–2 min. To entrap nanoceria in an agarose matrix, they were dissolved in sodium acetate buffer (100 mM, pH 5.3) at a concentration of 15 mg/mL and then mixed with 2% (*w*/*v*) agarose solution in a 1:4 volume ratio. After mixing rigorously, 15 mL of the mixture was poured into a petri dish (90 × 15 mm, SPL Lifesciences, Pocheon, Korea) and left to solidify at room temperature (RT) for 1 h. Next, the agarose gel was imprinted with a 200 μL pipet tip as the mold, to make a tablet-type agarose_nanoceria (5.4 mm in diameter), which fitted perfectly into the wells of a 96-well plate. An agarose composite with embedded Gal Ox and nanoceria (agarose_nanoceria + Gal Ox) was prepared by mixing nanoceria (15 mg/mL in sodium acetate buffer (100 mM, pH 5.3), Gal Ox (6 mg/mL in sodium acetate buffer (100 mM, pH 5.3), and 2% agarose solution in a 1:1:3 volume ratio and following the same procedure as that described for agarose_nanoceria preparation. 

The agarose_nanoceria or agarose_nanoceria + Gal Ox tablets were washed with deionized water three times and then used in further experiments. The amounts of protein and nanoceria that leached from the agarose composite into the supernatant were measured using the BCA protein assay (PIERCE, Rockford, IL, USA) and inductively coupled plasma atomic emission spectroscopy (ICP-AES) (Polyscan 60E, Thermo Jarrell, Franklin, MA, USA), respectively. The final concentrations of Gal Ox and nanoceria within the agarose composite were calculated using the differences between the initial and the leached concentrations. The prepared agarose composites were also analyzed by field emission TEM (Tecnai, FEI, Amsterdam, The Netherlands) in scanning transmission electron microscopy (STEM) and energy dispersive X-ray spectroscopy (EDS) imaging modes. For scanning electron microscopy (SEM) and Fourier transform infrared (FT-IR) analysis, the samples were freeze-dried for 2 days and then analyzed with a field emission scanning electron microscope (Magellan 400) and an FT-IR Spectrometer (Nicolet iS50, Thermo Fisher Scientific, Madison, WI, USA), respectively. The hydrodynamic diameters of free nanoceria were determined by dynamic light scattering (DLS) analysis using a Zetasizer Nano-ZS (Malvern Co., Worcestershire, UK).

### 2.3. Determination of H_2_O_2_ Using Agarose_Nanoceria

H_2_O_2_ concentration was determined using a transparent 96-well plate as follows. A mixture containing free nanoceria (0.2 mg) or an agarose_nanoceria tablet (one tablet approximately contains 0.2 mg nanoceria) and H_2_O_2_ (20 μL at varied concentrations) in 180 μL sodium acetate buffer (100 mM, pH 5.3) was incubated at RT for 10 min. The images of the resultant well-plate were then acquired using a smartphone (GALAXY S8 NOTE, Samsung, Seoul, Korea), and were subjected to quantitative image processing via conversion to “CMYK” mode using ImageJ software (version 1.48, NIH, Bethesda, MD, USA).

### 2.4. Determination of Galactose Using Agarose_Nanoceria + Gal Ox

Galactose concentration was determined using a 96-well plate as follows. A mixture containing agarose_nanoceria + Gal Ox (one tablet approximately containing 0.2 mg nanoceria and 0.08 mg Gal Ox) and galactose (20 μL at diverse concentrations) in 180 μL sodium acetate buffer (100 mM, pH 5.3) was added into the wells of a 96-well plate and incubated at 37 °C for 30 min. The resultant well-plate was directly used to obtain images with a smartphone, and other procedures were the same as those used for H_2_O_2_ determination.

Long-term stability of the agarose_nanoceria + Gal Ox tablets was examined in aqueous buffer (100 mM sodium acetate, pH 5.3) at RT under static conditions, by measuring color intensity in response to 10 mM galactose at predetermined time points. The relative color intensity (%) was calculated using the ratio of the residual color intensity to the original one. Synthetic reproducibility of the agarose_nanoceria + Gal Ox tablets was also assessed by measuring the color intensities of the tablets toward 10 mM galactose, which were prepared from 5 different synthetic batches.

To determine the amount of galactose in human serum, the original concentration of galactose in serum was first determined using a galactose assay kit (Sigma-Aldrich). Pre-determined amounts of galactose were then added to human serum to get spiked samples that represent normal, boundary, and galactosemia levels of galactose. The concentration of galactose in each spiked sample (20 μL) was measured using the same procedure as mentioned above. The precision and reproducibility of the assays were assessed by determining the recovery rate (recovery (%) = measured value/actual value × 100) and the coefficient of variation (CV (%) = SD/average × 100).

## 3. Results and Discussion

### 3.1. Construction of Agarose Composite for Colorimetric Determination of Galactose

**Figure 1:**
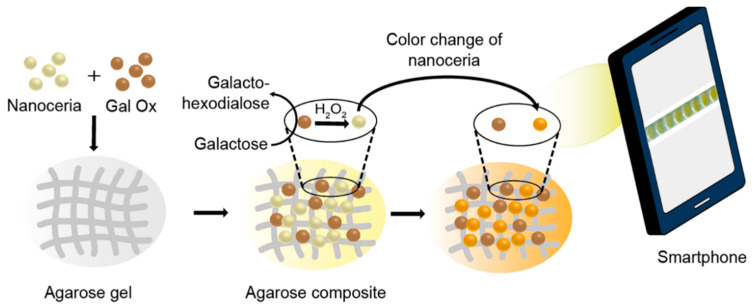
Schematic illustration of the agarose composite consisting of nanoceria and Gal Ox for the smartphone-mediated, reagent-free colorimetric determination of galactose.

A composite system for galactose quantification, containing nanoceria and Gal Ox simultaneously entrapped within an agarose gel matrix, was established (Figure 1). It was constructed by simply mixing nanoceria and Gal Ox in 2% (*w*/*v*) aqueous agarose solution, followed by gelation at RT for 1 h. This process resulted in about 18% and 8% loading for nanoceria and Gal Ox, respectively. We envisioned that the agarose composite (agarose_nanoceria + Gal Ox) would serve as an efficient colorimetric galactose biosensor capable of being used for diagnosing galactosemia, one of the major newborn metabolic disorders. In the presence of galactose, Gal Ox in the composite catalyzed the oxidation of galactose to produce H_2_O_2_, which subsequently interacted with the nanoceria to induce the oxidation of surface-exposed Ce^3+^ to Ce^4+^ species, and produced peroxide complexes at the nanoceria surface [18,19]. This promoted the vivid color change of nanoceria from white to intense yellow without any chromogenic substrate. Images were acquired using a smartphone under laboratory conditions, and quantitative information was obtained by simple image processing by converting the obtained JPEG-type image to “CMYK” mode using ImageJ software.

**Figure 2:**
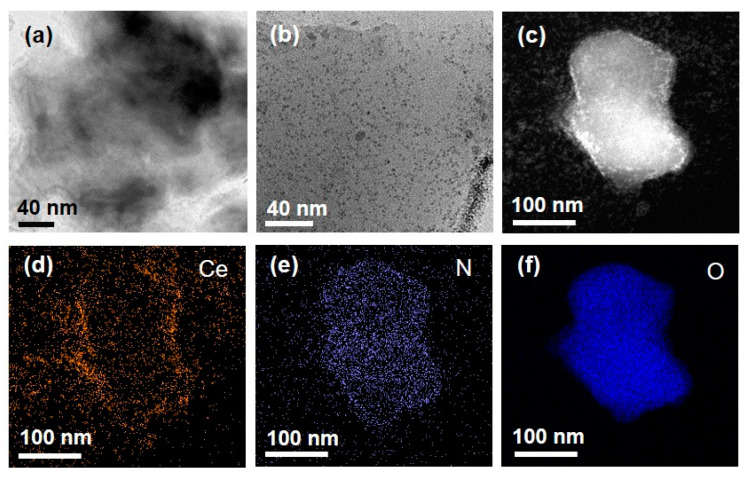
TEM images of (**a**) free agarose gel and (**b**) agarose_nanoceria. (**c**) STEM images of agarose composite entrapping both nanoceria and Gal Ox, and the corresponding EDS maps of (**d**) Ce, (**e**) N, and (**f**) O elements.

**Figure 3:**
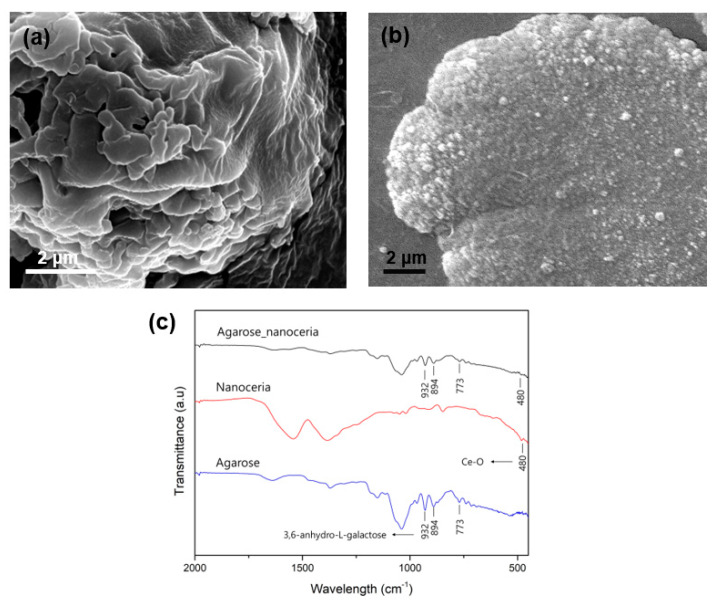
SEM images of (**a**) free agarose gel and (**b**) the agarose_nanoceria mixture. (**c**) FT-IR spectra of agarose_nanoceria, nanoceria, and agarose.

To obtain structural insights for the synthesized agarose composites, TEM analyses coupled with EDS elemental mapping were performed (Figure 2). The images revealed that the nanoceria were incorporated within the hydrogel. Elemental mapping images of Ce, N, and O elements, which are the main components of nanoceria, Gal Ox, and agarose, respectively, demonstrated that Gal Ox was homogeneously distributed throughout the agarose gel matrix. Nanoceria seemed to prefer adsorption on the rough and porous gel surfaces to their dispersion; however, there were still many nanoceria observed throughout the gel matrix, which may contribute to an enhanced colorimetric response compared with that of free nanoceria, which are prone to aggregation when suspended in an aqueous solution [20]. SEM images also showed the existence of nanoceria within the agarose gel, and their porous surfaces (Figure 3a,b), which may facilitate the transfer of galactose molecules through the gel matrix. Further TEM and SEM images are also shown in Figure S1. We also performed FT-IR analysis to analyze and compare the chemical structures of agarose_nanoceria with those of nanoceria and agarose (Figure 3c). The peaks observed at 773, 894, and 932 cm^−1^ in the spectrums of both agarose and agarose_nanoceria were attributed to 3,6-anhydro-L-galactose, which is the skeletal structure of the agarose gel [21,22]. The characteristic peak at 480 cm^−1^ for both nanoceria and agarose_nanoceria was attributed to the Ce–O–O stretching vibration, clearly indicating that nanoceria were encapsulated in the agarose gel matrix [23,24].

### 3.2. Vivid Colorimetric Responses of Agarose_Nanoceria toward H_2_O_2_

**Figure 4:**
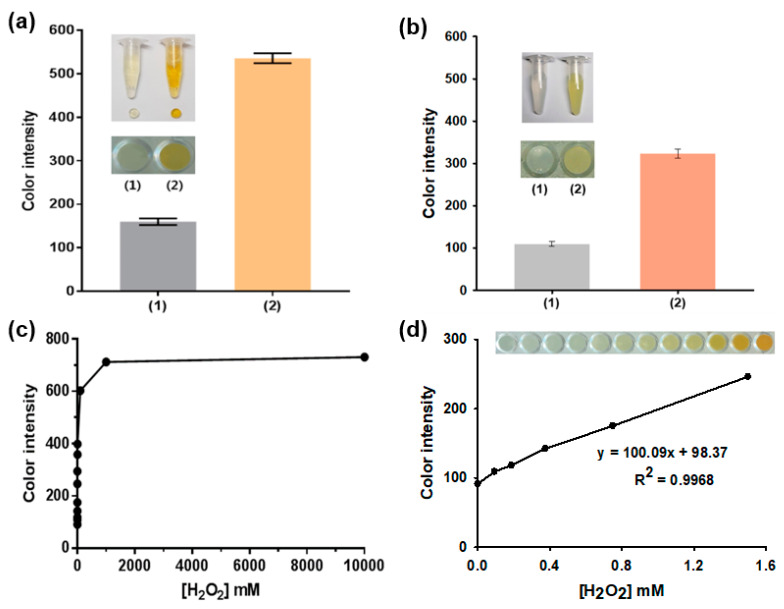
Colorimetric detection of H_2_O_2_ using agarose_nanoceria composite. Real images of the color-change reaction in the (1) absence and (2) presence of H_2_O_2_ (10 mM) and the corresponding color intensities observed using (**a**) agarose_nanoceria and (**b**) free nanoceria. (**c**) A dose-response curve for H_2_O_2_ determination using the agarose_nanoceria, and (**d**) the corresponding linear calibration plot with a real image. Each error bar represents the standard deviation of three independent measurements.

The colorimetric responses of agarose_nanoceria were examined in the presence of H_2_O_2_ and were quantified by capturing images with a smartphone (Figure 4). This assay is based on the principle that nanoceria develop a yellowish color proportional to the H_2_O_2_ concentration in a sample. Free nanoceria were used as the control. For image processing with the ImageJ software, the real images of agarose_nanoceria and free nanoceria were converted into the CMYK mode and analyzed. The investigations on the effects of buffer pH and composition, gel density, and temperature on the color intensity of agarose_nanoceria, showed that sodium acetate buffer (100 mM, pH 5.3), 2% (*w*/*v*) agarose, and 37 °C were ideal assay conditions (Figure S2). Although the incubation at 37 °C resulted in maximal color intensity, the incubation at RT was adopted rather than 37 °C because of the experimental convenience and the sufficiently acceptable sensing performance of agarose_nanoceria at RT (about 90% of maximum color intensity). Under the optimized conditions, the agarose_nanoceria enabled clear color change from white to intense yellow with 10 mM H_2_O_2_ during a 10 min reaction. The color change was similarly observed for free nanoceria as well; however, the intensity of the color developed was ~2-fold lower than that of agarose_nanoceria (Figure 4a,b), possibly due to the aggregated nature of free nanoceria [20], which is in contrast to the well-dispersed nature of nanoceria entrapped within the agarose matrix (Figure 2b). DLS analyses also showed that free nanoceria yielded ~300 nm of hydrodynamic diameter, while their single particle size was estimated to be only ~5 nm (Figure S3), confirming their aggregation in our assay conditions. Based on the enhanced color changes of agarose_nanoceria in the presence of H_2_O_2_, multiple samples containing various concentrations of H_2_O_2_ were analyzed (Figure 4c). The color intensity increased with increasing H_2_O_2_ concentrations, having a dynamic linear range of 0.1–1.5 mM (R^2^ = 0.9968), and the limit of detection (LOD) was calculated to be 0.017 mM (Figure 4d), which is enough for coupling with Gal Ox and creating an efficient galactose assay system. 

### 3.3. Analytical Capabilities of the Agarose Composite: Specificity, Linearity, Sensitivity, and Precision for Galactose Determination

**Figure 5:**
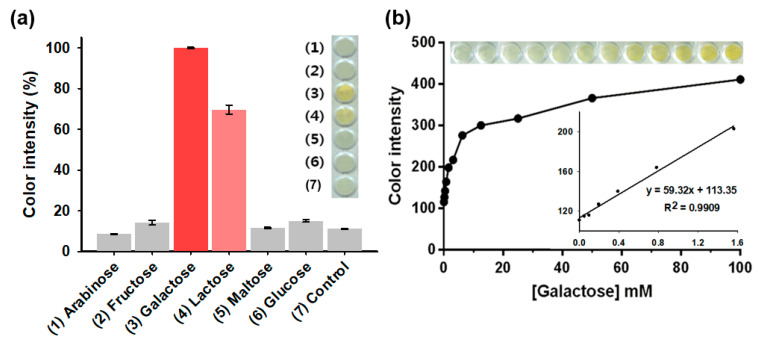
(**a**) Real images of the selective colorimetric detection of galactose using the agarose composite entrapping nanoceria and Gal Ox with the corresponding color intensities. A 5 mM concentration of galactose and lactose was used, while 50 mM of other carbohydrates was used in the experiments. (**b**) A dose-response curve, real images, and the corresponding linear calibration plots for galactose determination using the composite entrapping nanoceria and Gal Ox. Each error bar represents the standard deviation of three independent measurements.

Through the simple colorimetric reaction of agarose_nanoceria + Gal Ox without any colorimetric reagent, galactose was specifically determined as it showed a vivid color change from white to intense yellow in 30 min (Figure 5a). On the contrary, no significant color change was observed for the negative control samples, such as arabinose, fructose, maltose, and glucose, which confirmed the excellent specificity of the assay system towards the target galactose. As already known, Gal Ox shows significant reactivity toward lactose as substrate; there was a considerable signal detected for lactose in this study. However, this undesirable reaction would not interfere with our galactose determination assay, since lactose is always hydrolyzed into glucose and galactose by lactase in our body, and hence, lactose rarely appears in a considerable amount in human blood [25].

Through the analysis of dose-response curve, the LOD for galactose was determined to be as low as 0.05 mM, with a linear range from 0.1 to 1.5 mM (Figure 5b). The LOD value of the assay system was calculated by this formula: LOD = 3 × δ/slope, where δ is the standard deviation of blank and slope is the slope of calibration curve [26]. Considering that the cut-off value of galactosemia is about 0.44 mM (8 mg/dL), the current system is suitable to distinguish patients with galactosemia from normal persons [27]. Although several fluorescence-based enzymatic assays showed higher sensitivity, the LOD and linear range values of our system are among the best results describing colorimetric detection of galactose (Table S1). Furthermore, our assay system solely enables reagent-free colorimetric determination of galactose, which is quite advantageous in practical applications.

We generally used the agarose composites directly after the synthesis; however, they could be stored at 4 °C for one-month period of time without any significant loss of activity (data not shown). Then, the effects of entrapment of Gal Ox or nanoceria within agarose gel on stability were examined. To this, residual activities of the agarose_nanoceria + Gal Ox were determined along with those of free Gal Ox incubated in the presence of either agarose_nanoceria or free nanoceria. During 10 days of incubation at RT under static conditions, our agarose_nanoceria + Gal Ox almost retained their original activity, while free Gal Ox-mediated samples displayed significant losses of activity (Figure S4), showing the vivid enhancement in stability. Furthermore, agarose_nanoceria + Gal Ox composites, prepared from different synthetic batches, yielded similar color intensity with low variations, demonstrating the synthetic reproducibility (Figure S5).

Finally, we evaluated the diagnostic capability of the agarose_nanoceria + Gal Ox-based assay using clinical human serum samples containing levels of galactose corresponding to normal, boundary, and galactosemia (normal; ≤0.44 mM, boundary; 0.44–1.11 mM, and galactosemia; >1.11 mM) [28]. The original amount of galactose in the serum samples was first determined using a galactose assay kit, and a pre-determined amount of galactose was then added to establish the representative levels. According to the experimental results, the serum galactose levels were quantitatively determined with excellent precision, yielding CVs ranging from 4.80% to 6.90% and recoveries ranging from 94.47% to 105.86% (Table 1), demonstrating the excellent reproducibility and reliability of this assay. These results prove that the reagent-free colorimetric galactose assay described herein may serve as a promising analytical tool to diagnose galactosemia in clinical settings.

## 4. Conclusions

Herein, we prepared a composite system consisting of nanoceria and Gal Ox simultaneously entrapped within an agarose gel matrix as a reagent-free colorimetric sensor to detect galactose. The developed system yielded excellent selectivity, sensitivity, and linearity by processing the real image acquired on-site using a smartphone. The clinical application of the bioassay was successfully demonstrated by accurately determining the galactose concentrations from clinical human serum samples. Thus, this agarose composite-based galactose assay might serve as a promising analytical tool for a rapid, robust, and convenient method for galactose quantification, which is essential for the diagnosis of galactosemia in newborns. Since the current system enabled rapid visual detection of the target molecule in an instrumentation-free and cost-effective manner without the involvement of any chromogenic substrate, it should find practical applications in POCT environments.

## Figures and Tables

**Table 1:** Detection precision of the agarose_nanoceria_Gal Ox-based assay system for the determination of galactose levels in spiked human serum samples.

	Original Amount (mM)	Added Galactose Concentration (mM)	Expected Galactose Concentration (mM)	Measured ^a^ Galactose Concentration (mM)	SD ^b^	CV ^c^ (%)	Recovery ^d^ (%)
Normal	0.060	0.139	0.199	0.188	0.012	6.38	94.47
Boundary		0.589	0.649	0.687	0.033	4.80	105.86
High		1.333	1.393	1.377	0.095	6.90	98.85

^a^ The average value of five successive measurements. ^b^ The standard deviation (SD) of five successive measurements. ^c^ Coefficient of variation = (SD/mean) × 100. ^d^ Recovery = (measured value/expected value) × 100.

## Supplementary Materials

The following figures are available online at https://www.mdpi.com/2079-4991/10/5/895/s1. Figure S1: (a) TEM and (b) SEM images of agarose_nanoceria. Figure S2: Effects of (a) temperature, (b) buffer system (pH and composition), and (c) gel density on the level of color intensity for the detection of H_2_O_2_. Figure S3: Hydrodynamic size distributions of free nanoceria measured by DLS. Figure S4: Stability of agarose_nanoceria + Gal Ox and free Gal Ox in the presence of either agarose_nanoceria or free nanoceria. Figure S5: Color intensity toward 10 mM galactose using agarose_nanoceria + Gal Ox composites prepared from five different batches. Table S1: An overview on the essential assay attributes of optical determination of galactose.
