# Supplementary material for: Reagent-Free Colorimetric Assay for Galactose Using Agarose Gel Entrapping Nanoceria and Galactose Oxidase
Source: Nanomaterials (Basel). 2020 May 8;10(5):895. doi: 10.3390/nano10050895 (PMC7279418; doi:10.3390/nano10050895)
Supplement: Supplementary file 1 [file nanomaterials-10-00895-s001.pdf]

nanomaterials

Supplementary Materials

Reagent-Free Colorimetric Assay for Galactose Using Agarose Gel Entrapping Nanoceria and Galactose Oxidase

Phuong Thy Nguyen, Hee Tae Ahn and Moon Il Kim *

Department of BioNano Technology, Gachon University, 1342 Seongnamdae-ro, Sujeong-gu, Seongnam, Gyeonggi 13120, Korea; nnphuongthy18@gmail.com (P.T.N.); venice4@naver.com (H.T.A.)

* Correspondence: moonil@gachon.ac.kr; Tel.: +82-31-750-8563

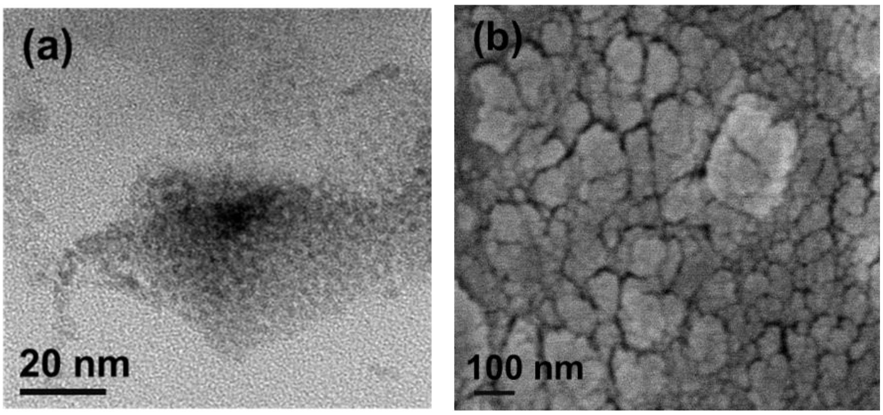

Figure S1. (a) TEM and (b) SEM images of agarose_nanoceria.

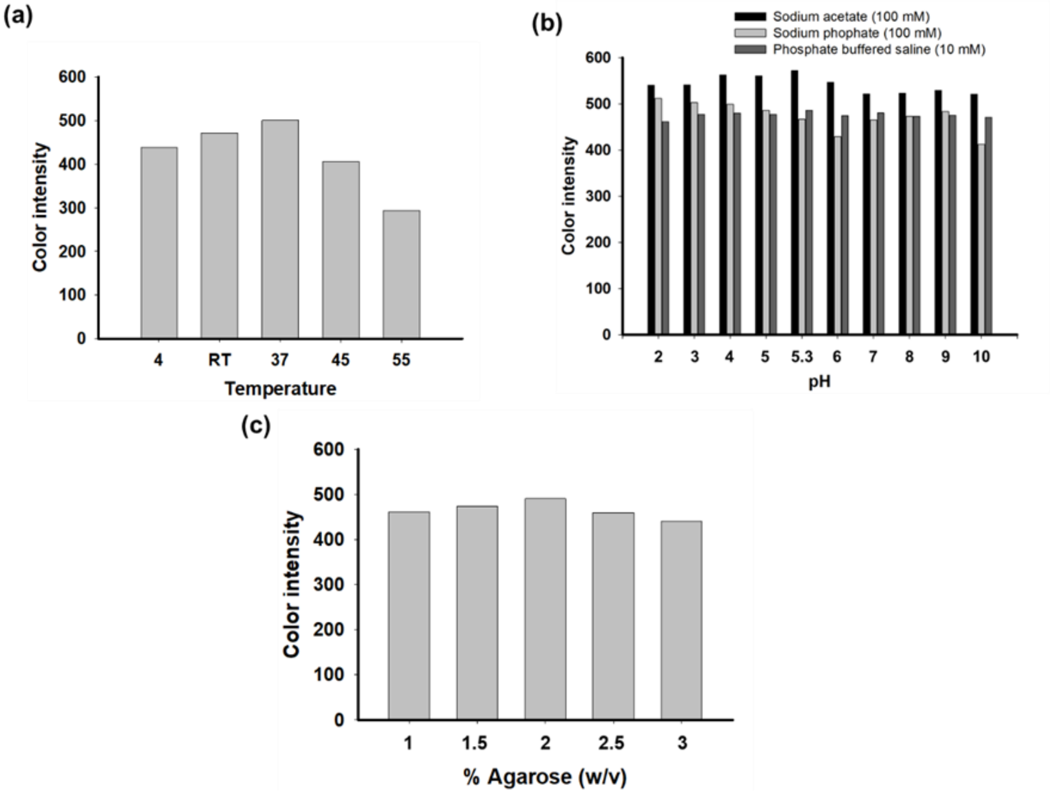

Figure S2. Effects of (a) temperature, (b) buffer system (pH and composition), and (c) gel density on the level of color intensity for the detection of H_2O_2.

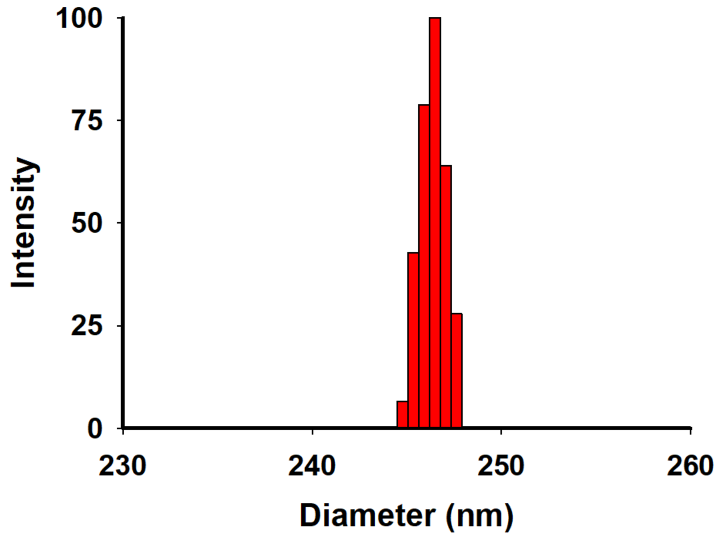

Figure S3. Hydrodynamic size distributions of free nanoceria measured by DLS.

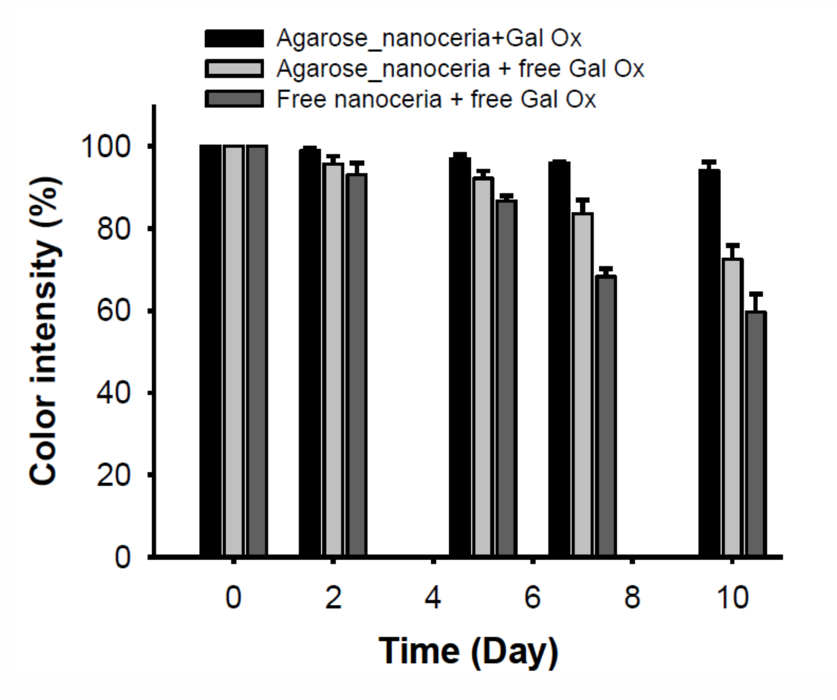

Figure S4. Stability of agarose_nanoceria+Gal Ox and free Gal Ox in the presence of either agarose_nanoceria or free nanoceria. Samples were incubated in aqueous buffer (100 mM sodium acetate, pH 5.3) at RT under static conditions and the error bars represent the standard deviation of three independent measurements.

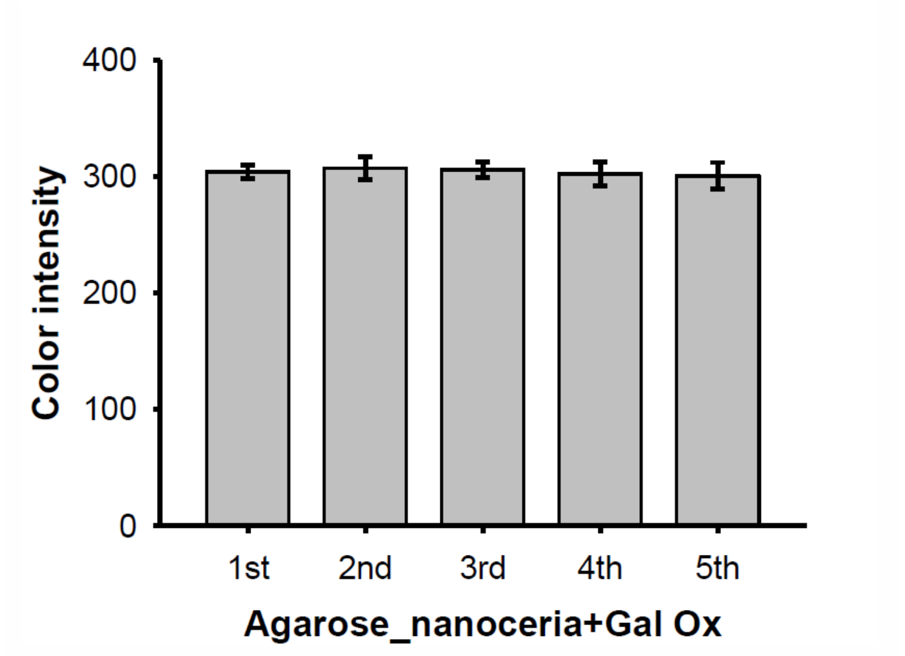

Figure S5. Color intensity toward 10 mM galactose using agarose_nanoceria+Gal Ox composites prepared from 5 different synthetic batches.

Table S1. An overview on the essential assay attributes of optical determination of galactose.

Required reagent	Detection method	Reaction time (h)	Linear range (μM)	LOD (μM)	Estimated cost	Stability	Ref.
Resazurin	Fluorescence	4	1-1000	5	High	Not tested	[S1]
Amplex UltraRed	Fluorescence	0.5	2-80	2	Medium	Not tested	[S2]
3,3',5,5'-tetramethylbenzidine (TMB)	Colorimetry	3	10-20,000	3	Low	Not tested	[S3]
2-(p-iodophenyl)-3-(p-nitrophenyl)-5-phenyltetrazolium chloride (INT)	Colorimetry	0.75	27.8-2780	27.8	Medium	Not tested	[S4]
2,20-Azino-bis(3-ethylbenzo-thiazoline-6-sulfonic acid) diammonium salt (ABTS)	Colorimetry	0.67	55.6-1111	27.8	Low	~20 days	[13]
Reagent-free	Colorimetry	0.5	100-1500	50	Low	~10 days	This work

References

S1. Shapiro, F.; Silanikove, N. Rapid and accurate determination of D-and L-lactate, lactose and galactose by enzymatic reactions coupled to formation of a fluorochromophore: Applications in food quality control. *Food Chem.* **2010**, *119*, 829–833.

S2. Liu, C.H.; Tseng, W.L. Oxidase-functionalized Fe_3O_4 nanoparticles for fluorescence sensing of specific substrate. *Anal. Chim. Acta* **2011**, *703*, 87–93.

S3. Li, M.; Yang, J.; Ou, Y.; Shi, Y.; Liu, L.; Sun, C.; Zheng, H.; Long, Y. Peroxidase-like activity of 2′,7′-difluorofluorescein and its application for galactose detection. *Talanta* **2018**, *182*, 422–427.

S4. Kianmehr, A.; Mahrooz, A.; Ansari, J.; Oladnabi, M.; Shahbazmohammadi, H. The rapid and sensitive quantitative determination of galactose by combined enzymatic and colorimetric method: application in neonatal screening. *Appl. Biochem. Biotechnol.* **2016**, *179*, 283–293.

© 2020 by the authors. Submitted for possible open access publication under the terms and conditions of the Creative Commons Attribution (CC BY) license (http://creativecommons.org/licenses/by/4.0/).
